# Feasibility of Race by Sex Intersectionality Research on Suicidality in the Adolescent Brain Cognitive Development (ABCD) Study

**DOI:** 10.3390/children8060437

**Published:** 2021-05-23

**Authors:** Shervin Assari, Shanika Boyce, Mohsen Bazargan

**Affiliations:** 1 Department of Family Medicine, Charles Drew University, Los Angeles, CA 90059, USA; Mohsenbazargan@cdrewu.edu; 2 Department of Urban Public Health, Charles Drew University, Los Angeles, CA 90059, USA; 3 Department of Pediatrics, Charles Drew University, Los Angeles, CA 90059, USA; shanikaboyce@cdrewu.edu; 4 Department of Family Medicine, University of California, Los Angeles (UCLA), Los Angeles, CA 90095, USA

**Keywords:** sex, race, suicidality, suicide, children

## Abstract

Intersectional research on childhood suicidality requires studies with a reliable and valid measure of suicidality, as well as a large sample size that shows some variability of suicidality across sex by race intersectional groups. Objectives: We aimed to investigate the feasibility of intersectionality research on childhood suicidality in the Adolescent Brain Cognitive Development (ABCD) study. We specifically explored the reliability and validity of the measure, sample size, and variability of suicidality across sex by race intersectional groups. Methods: We used cross-sectional data (wave 1) from the ABCD study, which sampled 9013 non-Hispanic white (NHW) or non-Hispanic black (NHB) children between the ages of 9 and 10 between years 2016 and 2018. Four intersectional groups were built based on race and sex: NHW males (*n* = 3554), NHW females (*n* = 3158), NHB males (*n* = 1164), and NHB females (*n* = 1137). Outcome measure was the count of suicidality symptoms, reflecting all positive history and symptoms of suicidal ideas, plans, and attempts. To validate our measure, we tested the correlation between our suicidality measure and depression and Child Behavior Checklist (CBCL) sub-scores. Cronbach alpha was calculated for reliability across each intersectional group. We also compared groups for suicidality. Results: We observed some suicidality history in observed 3.2% (*n* = 101) of NHW females, 4.9% (*n* = 175) of NHW males, 5.4% (*n* = 61) of NHB females, and 5.8% (*n* = 68) of NHB males. Our measure’s reliability was acceptable in all race by sex groups (Cronbach alpha higher than 0.70+ in all intersectional groups). Our measure was valid in all intersectional groups, documented by a positive correlation with depression and CBCL sub-scores. We could successfully model suicidality across sex by race groups, using multivariable models. Conclusion: Given the high sample size, reliability, and validity of the suicidality measure, variability of suicidality, it is feasible to investigate correlates of suicidality across race by sex intersections in the ABCD study. We also found evidence of higher suicidality in NHB than NHW children in the ABCD study. The ABCD rich data in domains of social context, self-report, schools, parenting, psychopathology, personality, and brain imaging provides a unique opportunity to study intersectional differences in neural circuits associated with youth suicidality.

## 1. Background

Childhood suicidality has been historically seen as a rare event [1,2,3]. As a result, studying sub-population variation in childhood suicidality requires large sample sizes and is rarely done [1,2,3]. This is one reason there is a paucity of research on the intersection of race and sex in children’s suicidality [1,2,3]. Thus, knowledge is minimally available on the suicidality of race by sex intersectional groups of children in the US. 

Traditionally, suicidality is seen as a non-Hispanic white (NHW) male problem [4,5]. This observation is based on the published literature suggesting that suicidality is higher in males than females [6,7,8] and in NHWs than non-Hispanic blacks (NHBs) [9,10,11]. Historically, suicide has not been accepted by the NHB culture [12,13]. Other reasons suicide has been low in NHB individuals have been high religiosity [12] and social support [14,15,16]. Besides, compared to NHWs, NHBs show higher levels of resilience and preparedness to handle new stressors as they emerge, in part because stress is everything but new to NHBs [17]. This NHB lower depression and suicidality than NHWs is also described as the NHB-NHW mental health paradox or NHB mental health advantage [18].

A study of 319,342 sex by race variation in suicidality of students in the American College Health Association-National College Health Assessment from 2011 to 2015 were compared. The results showed that from 2011 to 2015, suicidal ideation increased, males had higher suicidal ideation and attempts than females, and NHBs had a higher risk of suicidal ideation and attempts than NHWs. The study showed a link between suicidality and poor academic performance, weight-related problems (obesity), lack of physical activity, and poor sleep quality (insufficient sleep and sleep difficulties). This study questions the assumed NHB advantage in suicidality (lower than NHWs) among college students [19]. In another study of 229 18–33-year-old young adults, racial groups differed in the factors that precipitated their suicidality [20]. While other racial and ethnic groups such as Hispanic and Asian young adults were more likely than NHWs to report interpersonal stressors as a precipitating factor of their suicidality, NHB young adults were less likely than NHWs to report suicidality due to interpersonal stress [20].

Suicide is, however, no more an NHW male phenomenon. The historically low suicidality of NHBs has changed over the past years. Recent reports have warned about an unprecedented increase of suicidality in NHB boys, an increasing trend steeper than the NHW boys [21]. In response, the Congressional Black Caucus and the National Institute for Mental Health (NIMH) recently called the NHB youth suicide a national crisis.

Another construct other than race that strongly influences suicidality is sex. While suicidal ideation has been more common in females [22,23,24], suicidal attempt tends to be more common among males than females [25]. This is in part because suicidal behaviors may be a sub-domain of externalizing behaviors [3], which are more common in males than females [25]. Suicidality tends to be comorbid with other externalizing behaviors such as substance use, aggression, conduct disorder, behavioral problems, aggression, and homicide [26,27].

Still, very little is known on race by sex intersectional differences in children’s suicidality. Some research has suggested that the male-to-female ratio of suicidality differs across racial/ethnic groups [6,8,9,28,29,30]. However, we know more about death by suicide rather than suicidal ideation [31]. For example, the male-to-female completed suicide ratio was highest among NHB youth (more than 6 to 1) and lowest among Asian American youth (about 3:1) [31]. Although these findings suggest that sex and race/ethnicity may interact and have inter-dependent rather than independent effects, very little research has compared race/ethnicity groups for the effect of sex on suicidality) [31]. 

Having a valid tool and a diverse sample that enables us to study how race and sex intersectional groups differ in their suicidality may contribute to suicide prevention, diagnosis, and treatment across diverse groups. Similarly, such a tool and sample may inform us about how we should tailor interventions for each group. Finally, it will suggest which social group requires more immediate attention in terms of suicide prevention. Literature does not currently have such information; thus, it is difficult to tailor programs for suicide prevention across diverse groups of children [20].

The Adolescent Brain Cognitive Development (ABCD) study is the largest brain imaging study of pre-adolescents in the US. This study has sampled 11,000+ 9–10-year-old children from 21 study sites across 15 states and will follow them for up to 10 years as they transition from childhood to adolescents to young adulthood. This study has provided an unprecedented opportunity to investigate neurocircuits of suicidality across intersectional groups in US children [32,33].

### Objectives

We explored the feasibility of race by sex intersectionality research of suicidality in the Adolescent Brain Cognitive Development (ABCD) study baseline data [32,33]. We define feasibility based on considerable sample size, the presence of a reliable and valid suicidality measure, acceptable variation of suicidality, and ability to model variation across all race by sex intersectional groups. 

## 2. Methods

### 2.1. Design and Setting

We used the Adolescent Brain Cognitive Development (ABCD) study data [32,33]. Analysis used the wave 1 (baseline) of the data; thus, the study design was cross-sectional. The ABCD is a national study with a large, diverse sample of 9/10-year-old American children. Data collection of the ABCD baseline and recruitment of the participants were completed between years 2016 and 2018.

### 2.2. Sample and Sampling

The ABCD sample at baseline is composed of children who were between 9 and 10 years old. The ABCD sample was recruited from multiple cities that were nested to states. Most recruitments happened from the US school system [34]. In the current study, we only included NHW and NHB females and males.

### 2.3. Suicidality Measure

The children’s suicidality was measured by a scale that counted the number of symptoms and histories of suicide in the past and present. This measure was calculated based on an interview that measured various aspects of suicidality in participants. Participants’ responses were yes or no to various aspects of suicidality, as shown in Table A1. Our original suicidality measure provided two measures, first a count measure with a potential range between 0 and 22. Second, a dichotomous variable for any suicidal history. We, however, dropped some numbers due to no variation and enhancement of the reliability. Items used for our final measure are shown in Appendix A. These items are a part of the Kiddie Schedule for Affective Disorders and Schizophrenia (K-SADS) measure, which is a structured interview for psychiatric evaluation and clinical assessment. This measure is frequently applied in clinical and research settings and generates valid and reliable measurements [35,36]. 

### 2.4. Intersectional Groups

Study groups were built based on race and sex, and race. Of all participants, 3158 were NHW females, 3554 were NHW males, 1137 were NHB females, and 1164 were NHB males.

### 2.5. Covariates

Our covariates included age, household size, parental education, and neighborhood-level median home value. Age was recorded in months and was treated as a continuous measure. Household size was a continuous measure ranging between 2 and 19. Parental education, a continuous variable, ranged from 1 to 23, where a high score indicated high educational attainment of parents. Area-level median home value was already available in the ABCD data files. 

### 2.6. Validation Measures

In this study, we used the Child Behavior Checklist (CBCL) and history of K-SADS-based depression to validate our suicidality measure. CBCL is one of the most widely used measures of internalizing and externalizing problems. In this version, the parents report problem behaviors in their children. CBCL is commonly used in both research and clinical settings and has been translated into 90+ languages. The history of depression was measured using K-SADS, which is a validated measure.

### 2.7. Data Analysis

SPSS was used to analyze the data which were downloaded from the National Institute for Health (NIH) National Data Archive (NDA) website. Mean, standard deviation (SD), frequency, and relative frequency (%) were described overall and across race by sex groups. We used ANOVA and chi-square to compare the study variables among race by sex groups. We also calculated the reliability of our measure. For validity, we tested correlations between our suicidality measure and depression as well as CBCL scores. We also ran Poisson regression models to test if we can model the variability of suicidality in our sample. The ABCD participants were nested to families who were themselves nested to study sites. Thus, we calculated the intra-class correlation for our study outcome (suicidality). Our calculation showed an intra-class correlation of less than 0.05, which is negligible. As such, we did not apply mixed-effect or random effect models. 

### 2.8. Ethics

The ABCD study protocol received an Institutional Review Board (IRB) approval from several institutions, including but not limited to the University of California, San Diego (UCSD). All participating children provided assent. All participating parents signed an informed consent [37]. Our study was exempt from a full IRB review.

## 3. Results

### 3.1. Descriptive Data overall and across the Intersectional Group

A total number of 9013, NHW or NHB 9/10-year-old children entered our analysis. Of all participants, 3158 were NHW females, 3554 were NHW males, 1137 were NHB females, and 1164 were NHB males (Table 1).

Table 1 also summarizes the descriptive data by race and sex. While age and household size did not differ across groups, socioeconomic status (SES) was lower in NHB male and female than NHW male and female children. Suicidality was also higher in NHB male and female children. 

As Table 1 shows, our suicidality measure showed some variation in all groups, including 3.2% (*n* = 101), 4.9% (*n* = 175), 5.4% (*n* = 61), and 5.8% (*n* = 68) in NHW females, NHW males, NHB females, and NHB males, being suicidal at some time in their life, respectively.

### 3.2. Reliability

We found alpha Cronbach 0.70+ or more for our suicidality measure in NHW male and female children as well as NHB male and female children (Table 2). 

### 3.3. Validity

We found evidence for the validity of our suicidality measure overall and in NHW male and female children and NHB male and female children as there was a positive correlation between suicidality measure and depression and CBCL domains (Table 3). 

### 3.4. Race and Sex Comparison of Suicidality

In the pooled sample and in the NHB and NHW males and females, we had a considerable number of children with positive suicidality, enabling us to model the variability. Suicidality was higher in NHBs than NHWs and was lower in high SES families. We also found that NHB and males had higher suicidality (0/1) than NHW and females. The associations between race and sex remained significant after controlling for SES indicators (Model 2). High neighborhood SES and high family SES were both associated with less suicidality in the pooled sample (Table 4).

## 4. Discussion

Our analysis of ABCD data shows that it is feasible to study intersectional differences in children suicidality in the ABCD because, (1) a large n across all intersectional groups, (2) acceptable variability of suicidality in race by sex groups, and (3) high reliability and validity of our suicidality measure in all intersectional subgroups, and (4) considerable variability of the outcome, and (5) possibility of modeling the suicidality. We also observed that higher suicidality in NHB than NHW children. 

Our results are against the belief that (1) suicidality is very rare at ages 9 and 10, so we would not be able to find appropriate data sets with valid measures, large n, and enough distribution. This is particularly important because ABCD study has collected brain imaging and psychopathology data so we will be able to compare race by sex groups for correlates of suicidality. Our study confirms the feasibility of suicidality research in the intersectional groups of children in the ABCD study. As ABCD project has also collected extensive contextual and substance use data, it becomes a unique opportunity to study the intersectionality of suicide by race and sex, from age 9–10, forward. Researchers should not assume that suicide is too rare in ages 9–10, there would be no variation, so it would be impossible to model suicidality of children at this age across intersectional groups. 

We found higher suicidality in NHB than NHW children. Our finding is in line with the new trends on NHB youth suicide crisis. Unfortunately, NHB children suicidality is overlooked. Suicide is increasing in NHB children and youth [38]. As a result, there is a need to address NHB youth suicide crisis [21].

ABCD study provides an excellent opportunity to study intersectional variation in social, psychological, and biological pathways to children suicidality. Previous research has shown NHW and NHB differences in correlates of suicide. For example, parents’ education shows larger protective effects for NHW than NHB children, youth, and adults [39]. This is because socioeconomic status indicators may lose some of their effects due to structural racism [40]. These diminished effects of SES on suicide and other high-risk behaviors in NHB than NHW people are attributed to structural racism and segregation. As a result of racism, high SES NHB families continue to experience much stress, while high SES NHW families report very low stress across domains [41]. Similarly, high SES NHW families have financial security and wealth, while high SES NHB families have far less financial security and wealth [42].

Ali and colleagues used de-identified National Violent Death Reporting System (NVDRS) Restricted Access Database files from 2006–2015 to compare NHB-NHW differences in triggers of suicidality. All participants were 10-year or older. The authors used logistic regression analysis and revealed racial differentials in how non-alcohol substance abuse problems, intimate partner problems, and physical health problems correlated with the suicide of race by sex groups. These differences were not due to potential confounders. Authors advocated for attention to intersectional groups of race by sex for suicide prevention [43].

In one study [44], 5388 NHW and 759 NHB suicidal attempts resulting in hospitalization were compared. Sociodemographic factors, medical history, psychiatric disorders, and outcome (death) were compared across racial groups. The study did not find NHB-NHW differences in age, sex, or psychopathology, but NHBs and NHWs varied by insurance type. While NHWs were more likely to have private health insurance while NHBs were commonly under governmental insurance. NHBs with suicide attempts were more likely to be obese, while NHW participants who were suicidal were likely to be underweight. Physical health also shows a different profile across NHB and NHW suicidal attempts, with NHBs having more health problems than NHWs. The study did not find any differences in in-patient death rates of attempted suicide between NHW and NHB who attempted suicide [44]. 

The study had several limitations. First, the design was cross-sectional, which does not allow causal inferences. We did not study a wide range of confounders. For example, sexual orientation and gender identity and a history of sexual assault, a victim of bullying, depression, alcohol use, cigarette smoking, drug use, and prescription medication misuse are among predictors of suicidal behaviors in children and youth [30]. We also did not include other racial and ethnic groups such as Latino, Asian American, or Native Americans.

The results may have some implications. Clinical practice, public health service, and policy can use these findings. One size does not fit all, and sex differences (male dominance). In suicidality seems to be more relevant to NHW than NHB children. Tailored interventions and services across of race by sex intersectional groups may have some advantages, mainly because research has shown race by sex differences in suicidality correlations [43]. Joe conducted a review and found very few evidence-based suicide prevention or treatment strategies for the race by sex intersectional groups such as NHB male youth. They, however, mentioned that crossover effects for multisystemic therapy for reducing the risk for suicide ideation and attempts in NHB males might exist. That paper suggested that attachment-based family therapy is an important element of NHB males’ suicide prevention [45].

Higher suicidality of NHB than NHW children is concerning. The traditional assumption that males have a higher suicidality rate and suicide prevention is less relevant to NHB children is not accurate. This means that both NHB boys and girls are at suicidality risk. Similarly, NHB children are not at less risk, but more risk compared to NHW children. That means suicidality patterns of children are not shaped by their sex *or* race but their race and sex. More research is needed on how these patterns vary across age groups, how these differences emerge over time. It is unknown how intersectional groups differ in the mechanisms and methods of suicide. 

Interventions to prevent NHW and NHB youth’s suicidality should be ongoing, and policymakers should not assume that one size fits all. Comprehensive suicide prevention efforts should consider how race and sex correlate with children’s suicidality and may leverage such information to target their populations. More research is needed to develop interventions that leverage protective factors. Such research may help us with innovative prevention strategies that minimize the chance of lives lost due to suicide. As race distribution varies in different communities, and as race by sex groups differ in correlates of suicide, suicide prevention may be improved in the context of race and sex differences.

The ABCD study, however, is specifically designed and performed in US. As such, the results may be more relevant to US than any other region. The role of race and sex may differ across societies, so the results may not necessarily apply to European, Asian, African, or North American countries. In addition, this study is relevant to a high-income developed country and the patterns observed may not be necessarily relevant to developing or Lower-Middle Income countries (LMIC’s).

## 5. Conclusions

ABCD study has a large sample of 9/10-year-old American children, with a reliable and valid measurement of suicidality that can be successfully used to compare intersectional groups of children based on race and sex in suicidality. An intersectional study of suicidality in race by sex intersectional groups can be done at ages 9/10 using ABCD study. Now that ABCD has a valid/reliable measure and has statistical power to model suicidality across each intersectional group, the next step is to study race by sex variation in biological, psychological, and social mediators and moderators of suicidality in US children. Given the longitudinal design of this study, such intersectional groups can be followed for trajectories of suicidality over time.

## Figures and Tables

**Table 1:** Descriptive statistics by the intersection of race and sex.

	NHW Female		NHW Male		NHB Female		NHB Male	
	Mean	SD	Mean	SD	Mean	SD	Mean	SD
Age (Month)	9.48	0.50	9.50	0.51	9.45	0.51	9.49	0.52
Household Size	4.68	1.35	4.76	1.42	4.66	1.74	4.69	1.76
Parental Education *	17.61	1.95	17.51	2.02	15.37	2.59	15.39	2.53
Median Area-Level Home Value	2.86	1.83	2.88	1.86	1.53	1.23	1.54	1.27
	*n*	%	*n*	%	*n*	%	*n*	%
Suicidality (Any) *								
No	3057	96.8	3379	95.1	1076	94.6	1096	94.2
Yes	101	3.2	175	4.9	61	5.4	68	5.8

Non-Hispanic white (NHW); non-Hispanic black (NHB); standard deviation (SD); * *p* < 0.05.

**Table 2:** Reliability of our suicidality measure in American children by race and sex.

	All	Females	Males	NHW	NHB	NHW Female	NHW Male	NHB Female	NHB Male
Cronbach Alpha Full Measure	0.726	0.709	0.736	0.725	0.729	0.690	0.739	0.731	0.726
Cronbach Alpha After First Item Omitted	0.748	0.733	0.756	0.750	0.744	0.724	0.760	0.741	0.746

Non-Hispanic white (NHW); non-Hispanic black (NHB).

**Table 3:** Validity of suicidality measure in American children by race and sex.

	1	2	3	4	5	6	7	8	9	10
**NHW Female**										
1 Suicidality		0.13**	0.10 **	0.11 **	0.05 **	0.11 **	0.09 **	0.10 **	0.12 **	0.11 **
2 CBCL Total			0.74 **	0.59 **	0.56 **	0.73 **	0.70 **	0.59 **	0.86 **	0.80 **
3 CBCL Anxious Depressed				0.51 **	0.40 **	0.52 **	0.50 **	0.33 **	0.49 **	0.50 **
4 CBCL Withdrawal Depressed					0.33 **	0.45 **	0.40 **	0.33 **	0.42 **	0.43 **
5 CBCL Somatic Complaints						0.35 **	0.38 **	0.26 **	0.41 **	0.35 **
6 CBCL Social Problems							0.47 **	0.47 **	0.59 **	0.57 **
7 CBCL Thought Problems								0.35 **	0.63 **	0.49 **
8 CBCL Rule Breaking									0.52 **	0.55 **
9 CBCL Attention Problems										0.69 **
10 CBCL Aggressive Behaviors										
**NHW Male**										
1 Suicidality		0.11 **	0.07 **	0.09 **	0.05 **	0.09 **	0.08 **	0.11 **	0.10 **	0.10 **
2 CBCL Total			0.72 **	0.61 **	0.52 **	0.73 **	0.72 **	0.66 **	0.89 **	0.81 **
3 CBCL Anxious Depressed				0.53 **	0.38 **	0.51 **	0.51 **	0.36 **	0.49 **	0.49 **
4 CBCL Withdrawal Depressed					0.31 **	0.49 **	0.41 **	0.36 **	0.43 **	0.43 **
5 CBCL Somatic Complaints						0.35 **	0.37 **	0.28 **	0.38 **	0.34 **
6 CBCL Social Problems							0.51 **	0.48 **	0.60 **	0.55 **
7 CBCL Thought Problems								0.42 **	0.65 **	0.52 **
8 CBCL Rule Breaking									0.58 **	0.63 **
9 CBCL Attention Problems										0.71 **
10 CBCL Aggressive Behaviors										
**NHB Female**										
1 Suicidality		0.07 *	0.04	0.05	0.05	0.10 **	0.03	0.10 **	00.04	0.08 **
2 CBCL Total			0.77 **	0.63 **	0.59 **	0.78 **	0.72 **	0.72 **	0.90 **	0.85 **
3 CBCL Anxious Depressed				0.54 **	0.45 **	0.61 **	0.57 **	0.43 **	0.61 **	0.58 **
4 CBCL Withdrawal Depressed					0.35 **	0.53 **	0.47 **	0.41 **	0.48 **	0.51 **
5 CBCL Somatic Complaints						0.41 **	0.42 **	0.35 **	0.46 **	0.42 **
6 CBCL Social Problems							0.56 **	0.54 **	0.64 **	0.64 **
7 CBCL Thought Problems								0.50 **	0.68 **	0.58 **
8 CBCL Rule Breaking									0.66 **	0.67 **
9 CBCL Attention Problems										0.75 **
10 CBCL Aggressive Behaviors										
**NHB Male**										
1 Suicidality		0.08 **	0.12 **	0.09 **	0.06 *	0.08 **	0.07 *	−0.01	0.06	0.02
2 CBCL Total			0.80 **	0.65 **	0.57 **	0.83 **	0.79 **	0.74 **	0.93 **	0.87 **
3 CBCL Anxious Depressed				0.59 **	0.47 **	0.65 **	0.65 **	0.51 **	0.66 **	0.62 **
4 CBCL Withdrawal Depressed					0.37 **	0.56 **	0.56 **	0.37 **	0.52 **	0.51 **
5 CBCL Somatic Complaints						0.45 **	0.44 **	0.33 **	0.45 **	0.43 **
6 CBCL Social Problems							0.67 **	0.56 **	0.73 **	0.67 **
7 CBCL Thought Problems								0.53 **	0.73 **	0.65 **
8 CBCL Rule Breaking									0.67 **	0.76 **
9 CBCL Attention Problems										0.79 **
10 CBCL Aggressive Behaviors										

Child Behavior Checklist (CBCL); non-Hispanic white (NHW); non-Hispanic black (NHB); standard deviation (SD); * *p* < 0.05, ** *p* < 0.01.

**Table 4:** Association between race, sex, and suicidality in American children.

	B	SE	95% CI		*p*	B	SE	95% CI		*p*
	Model 1 All					Model 2 (M1 + SES Indicators)				
						All				
Race (Black)	0.40	0.07	0.26	0.55	<0.001	0.23	0.09	0.06	0.41	0.009
Sex (Male)	0.40	0.07	0.26	0.54	<0.001	0.45	0.08	0.30	0.60	<0.001
Age (Month)	0.01	0.07	−0.13	0.14	0.886	0.00	0.07	−0.14	0.14	0.998
Household Size						−0.10	0.03	−0.15	−0.04	<0.001
Parental Education						−0.05	0.02	−0.08	−0.01	0.006
Neighborhood Median Home Value						−0.03	0.02	−0.07	0.02	0.288

SES: Socioeconomic Status, B: Regression Coefficient, SE: Standard Error. Model 1: Only adjusted for demographic factors (SES not included), Model 2: Adjusted for demographic and SES factors.

## Data Availability

Data are available at https://nda.nih.gov/abcd (accessed on 5 May 2021).

## Appendix A

**Table A1:** Suicidality items in this study.

		Item	Timing	Distribution/Variance	In the Final Measure
Symptom 1	W1_ksads_23_148_t	Suicidal Ideation	Present	+	-
Symptom 2	W1_ksads_23_149_t	Suicidal Ideation	Past	+	+
Symptom 3	W1_ksads_23_150_t	Suicidal Attempt	Present	+	+
Symptom 4	W1_ksads_23_807_t	Self-injury, intent to die	Present	-	-
Symptom 5	W1_ksads_23_808_t	Self-Injury, thought could die from behavior	Present	+	+
Symptom 6	W1_ksads_23_809_t	Suicidal ideation thought of method	Present	+	+
Symptom 7	W1_ksads_23_810_t	Suicidal ideation, intent to act	Present	+	+
Symptom 8	W1_ksads_23_811_t	Suicidal ideation, specific plan	Present	+	+
Symptom 9	W1_ksads_23_812_t	Suicidal behavior, made preparations	Present	+	+
Symptom 10	W1_ksads_23_813_t	Aborted or interrupted suicide attempts	Present	+	+
Symptom 11	W1_ksads_23_814_t	Method of actual suicide attempt	Present	+	+
Symptom 12	W1_ksads_23_815_t	Suicide attempt, thought could die	Present	+	+
Symptom 13	W1_ksads_23_816_t	Self-injury, intent to die	Past	-	-
Symptom 14	W1_ksads_23_817_t	Self-Injury, thought could die from behavior	Past	+	+
Symptom 15	W1_ksads_23_818_t	Suicidal ideation thought of method	Past	-	-
Symptom 16	W1_ksads_23_819_t	Suicidal ideation, intent to act	Past	+	+
Symptom 17	W1_ksads_23_820_t	Suicidal ideation, specific plan	Past	+	+
Symptom 18	W1_ksads_23_821_t	Suicidal behavior, made preparations	Past	+	+
Symptom 19	W1_ksads_23_822_t	Aborted or interrupted suicide attempts	Past	+	+
Symptom 20	W1_ksads_23_823_t	Number of suicide attempts	Past	+	+
Symptom 21	W1_ksads_23_824_t	Suicide attempt, method	Past	+	+
Symptom 22	W1_ksads_23_825_t	Expect could die from suicide attempt	Past	+	+
